# Supplementary material for: The burden of out-of-pocket and indirect costs of cutaneous leishmaniasis patients in Minas Gerais, Brazil
Source: PLoS Negl Trop Dis. 2025 Apr 15;19(4):e0013020. doi: 10.1371/journal.pntd.0013020 (PMC12052389; doi:10.1371/journal.pntd.0013020)
Supplement: S1 File — (DOCX) [file pntd.0013020.s001.docx]

**Annex C - Expenses during treatment**

The questions below assess the impact of Cutaneus Leishmaniasis (CL) on your life. For each of the following questions, please indicate the answer that best describes your experiences.

Initials: _____________ Record no: _____________ Date: ___/___/____

**1) Family income pattern declared by the patient in one month:**

( ) < minimum monthly salaries *

( ) 1 minimum monthly salaries

( ) Up to minimum monthly salaries (up to R$2,424.00)

( ) 2 to 4 minimum monthly salaries (R$ 2,424.01 to R$ 4,848.00)

( ) 4 to 7 minimum monthly salaries (R$ 4,848.01 to R$ 8,484.00)

( ) 7 to 10 minimum monthly salaries (R$ 8,484.01 to R$ 12,120.00)

( ) 10 to 20 minimum monthly salaries (R$ 12,120.01 to R$ 22,240.00)

( ) Above 20 minimum monthly salaries (R$ 24,240.01 or more)

( ) Don't know

*If the family answered less than one minimum monthly salaries, they will be asked: What is the average amount your family receives in a month? ____________________________

NOTE: values in Real (BRL)

**What type of employment (tick as many options as necessary):**

( ) Employed (CLT/Contract/Public servant)

( ) Entrepreneur (self-employed)

( ) Pensioner

Note**:_______________________________________________________________________**

**2) Report of expenses during treatment observed by the patient (Period: start of treatment until the day of the interview)**

**a) Are there any transport costs to the Reference Center (bus/taxi/app/ambulance/petrol)?**

( ) Yes, What is the approximate cost?______________________________________________

( ) No

( ) Don't know

Note:________________________________________________________________

**b) How far is it in kilometres from the patient's home to the Reference Center** **?**

Note: ___________________________________________________

**c) Are there any food costs (snack/lunch/dinner/delivery fees)?**

( ) Yes, What is the approximate amount/day? __________________

( ) No

( ) Don't know

Note: ___________________________________________________________________

**d) Do you spend any money on delivery services not related to food?**

( ) Yes, What is the approximate amount and period? _________________________________

( ) No

( ) Don't know

Note: ___________________________________________________________________

**e) Are there any costs for overnight stays in hotels for the patient/family and/or carers?**

( ) Yes, What is the approximate amount and period? ____________________________

( ) No

( ) Cannot provide information

Note: ___________________________________________________________________

**f) Are there any costs for communication/internet/phone calls?**

( ) Yes, What is the approximate amount and period? ____________________________

( ) No

( ) Don't know

Note: ___________________________________________________________________

**g) Do you spend money on medicines not dispensed by SUS and purchased from a private pharmacy?**

( ) Yes, What is the approximate amount and period? ___________________________

( ) No

( ) Don't know

Note: ___________________________________________________________________

**h) Are there any costs for dressings/gauze/gloves/physiological solution?**

( ) Yes What is the approximate amount and period? ___________________________

( ) No

( ) Don't know

Note: ______________________________________________________________________

**i) There are costs for hiring a medical service to treat the condition (injections, dressings materials).**

( ) Yes, What is the approximate amount and period? ___________________________

( ) No

( ) Don't know

Note: ______________________________________________________________________

**j) Are there any costs for additional consultations (medical clinic/ ENT/ speech therapy/ psychology)?**

( ) Yes, What is the approximate amount and period? ___________________________

( ) No

( ) Don't know

Note: ______________________________________________________________________

**k) Are any tests paid for by the patient?**

( ) Yes, What is the approximate amount and period? __________________________

( ) No

( ) Don't know

Note: ______________________________________________________________________

**l) Are there any other sources of expenditure not described in this form?**

( ) Yes Cite: ________________________________________________________________ What is the approximate amount and period of expenditure? __________________________

( ) No

( ) Don't know

Note: ______________________________________________________________________

**3) Report of change in habits**

**a) Patient absences from work**

1. Did the patient miss work or stop earning any income during treatment?

( ) Yes* ( ) No ( ) Cannot say

2*. How many hours and number of times have you been absent in the last month? _____________________________________________________________________

**b) Companion**

1. Does the patient need a companion to attend appointments?

( ) Yes* ( ) No ( ) Cannot answer

2. Did the companion miss work or stop earning any income during treatment?

( ) Yes* ( ) No ( ) Can't say

3. How many hours has the CARER been absent and how many times in the last month?_____________________________________________________________

**c) Other help**

1. Did you ask a friend, family member or neighbour for help with a routine activity during your treatment?

( ) Yes ( ) No ( ) Can't say

If yes, how many hours a day?__________________________

1. Did you stop caring for a family member/dependent during your treatment (child/elderly/disabled/incapable)?

( ) Yes ( ) No ( ) Can't say

If yes, how many hours a day?_________________________.
