# Supplementary material for: The burden of out-of-pocket and indirect costs of cutaneous leishmaniasis patients in Minas Gerais, Brazil
Source: PLoS Negl Trop Dis. 2025 Apr 15;19(4):e0013020. doi: 10.1371/journal.pntd.0013020 (PMC12052389; doi:10.1371/journal.pntd.0013020)
Supplement: Attachment — Submitted filename: Response to Reviewers.docx [file pntd.0013020.s003.docx]

Dear Reviewers,

We are grateful for the careful review and suggestions that contributed to developing a more cohesive and higher-quality text.

We analyzed all aspects cited by the reviewer and answered, generating a new manuscript version. Highlight the changes in the manuscript, and responses to reviewers' comments are inserted in the table below, with details of the changes and responses requested.

We remain at your disposal for further clarification and await the article's definitive acceptance for publication.

Best Regards.

**Table 1 – Response to Reviewer 1**

| **Session** | **Comment** | **Response** |
| --- | --- | --- |
| **Methods** | The study is well designed and conducted. Patient's characteristics are well described. It is important to understand how the authors reached the sample size, it seems to be low and underpowered. Therefore, I ask the authors to explain it and consider that it may be a possible limitation to the study. Statistical analysis seems correct, no concerns about ethical or regulatory requirements. | Thanks for the careful review. The sample of patients was a prospective collection of all patients seen at the service over a 12-month period, so the sample size was not fixed, but depended on issues such as demand for the service, incidence of new cases of the disease, and capacity of the service. This information was added to the discussion of the article as a limitation of the sample (lines 358-360). |
| **Results** | The data is well presented but it would be important to show:  1. In Figure 1 or table 2: specific direct costs versus type of treatment.  2. In table 4: catastrophic family expenditure versus type of treatment. | This information is detailed in Table 3, which shows the direct costs, indirect costs, average family income and the average percentage of family spending for each type of treatment. This table has been supplemented with a description of catastrophic expenditures to make the data more complete and easier to visualise (Table 3). |
| **Conclusions** | Data are well discussed and the conclusions are well supported. Limitation due to sample size should be recognized.  Public relevance is well addressed and the paper will contribute to health care decision-making. | The sample size information was included in the discussion. |

**Table 2 – Response to Reviewer 2**

| **Session** | **Comment** | **Response** |
| --- | --- | --- |
| **Methods** | I have a question about why the data collection window appears to be between the first dose and 30 days after last dose. It was not clear whether the researchers go back to the same patient multiple times in that data collection period, or did they go only once? This is important to clarify, because if you are assessing costs only once at the beginning of the treatment, you may miss costs that occur later in the treatment (for example, adverse effects.) I appreciate that this is complicated slightly by the different duration of treatments for the different regimens.  The methods used otherwise appear to be well justified and appropriate, but I did have some questions about the analysis and the patients who were excluded from the study, which can be found in my comments in the editorial section.  The sample size itself was not very large (68), and there was only a single data point for some treatment regimens. In addition, getting data from multiple locations would have strengthened the results. But as a prospective study, this is fine. If possible, getting data from more locations, and for the different treatment regimens, would enable the running of more sophisticated analysis on this data set, including various regressions to control for and identify the most significant factors affecting the costs. | A treatment cycle for cutaneous leishmaniasis ranges from 10 to 30 days depending on each treatment, so this time window was defined so that all patients could answer the questionnaire about costs at the time they were already in treatment or shortly after completing it. This data collection was carried out only once and most often corresponds to the second clinic visit after the start of treatment, on average the fourteenth day of treatment. Some patients found it difficult to return halfway through treatment, so they answered the questions on their next visit to the clinic. Patients who were hospitalized for treatment (Lipossomal Anfotericin B) were interviewed at the end of treatment when they returned to the reference centre, thus justifying the 30 days after the last dose of the drug. We have gone into more detail in the methodology of the article to clarify these issues (Line 140-142). |
| **Results** | The small n in this case, restricts the analysis they could do here. Nevertheless, given the sample size, the results are well presented and clear to me.  There are some things that are interesting: for example, that ulcerated lesions have lower average costs than non-ulcerated lesions, but that may be due to the small sample size of non-ulcerated lesions.  Otherwise, the results, tables, and figures were clear to me. | Sample size information was included in the discussion with appropriate limitations (Line 358-360). |
| **Conclusions** | The discussion was appropriate and relevant. This would be good data for policy makers to see. I had some questions about things added to the discussion that were not apparent in the tables, but otherwise it looked good. | Thanks for the careful review. The answers to the other questions can be found in the lines below. |
| **Editorial and Data Presentation Modifications** | 1.) In the introduction section, please describe the typical diagnostic and treatment protocol in Brazil, so that it is easier to understand the duration of the diagnosis / treatment and understand what costs you are collecting? For example, if you are collecting cost data immediately after the first dose, you may miss adverse effects from the treatment that may appear later in the treatment cycle. | Information about the protocol in Brazil has been added to the introduction (Line 96-98). |
|  | 2,) Did the researchers go back to the same patient multiple times in that data collection period to collect cost data, or did they go only once? If they only surveyed a particular patient once for the costs, wouldn't the costs assessed after a first dose be different from the costs assessed after 20 doses? How would that affect your results? | As stated in the Methods section, cost data were collected through a single cross-sectional interview with each patient, conducted at various points within the treatment period. This approach means that cost estimates reflect different stages of the treatment journey, depending on when the interview took place (e.g., after the first dose, midway through treatment, or after completion). We acknowledge that this may introduce variability in the individual cost data, especially between patients interviewed early versus later in the treatment course. However, since our objective was to estimate the average cost per patient over the course of treatment, we considered the full treatment window—from the first dose to 30 days after the final dose—as the reference period. To address potential variability, we ensured that the sample included patients at different treatment stages, providing a more representative estimate of the overall cost pattern. information included in the article's methodology (Line 184-189). |
|  | 3.) How do the results change between the over and under 18 age groups? I suggest you separate the analysis for the over and under 18 age-groups, simply because the direct and indirect costs will likely be different for these two groups | There was only one patient under the age of 18, making it impossible to compare and define subgroups for this study. |
|  | 4.) Were there location specific factors (since all the data was from one testing centre), that may have influenced the results? | We believe that the local factors are related to the case profile, as this is a referral service that may concentrate more complex cases that are not diagnosed or treated in local services. Clinical questions strictly follow a protocol for which they are related to the clinical conditions of the patients and the local service network. |
|  | 5.) It is not clear what you mean when you say that "patients who had difficulty understanding the established criteria" were excluded from the study. Please explain this a little more? What criteria did they not understand? | Some illiterate patients, some patients with communication difficulties (hearing and speech), some patients with comprehension difficulties (frail elderly, disabled). We include this information in methods.  (Line 133-134). |
|  | 6.) Do you have any sociodemographic data about the excluded patients (those who decided not to participate)? For example, were they disproportionately female, or of a certain ethnicity or income group? If so, that limitation should be noted during the discussion. | Patients were approached at their first visit to the service and only those who agreed and signed the informed consent form proceeded with the study. The initial data collected were archived and not analysed in accordance with established ethical principles. |
|  | 7.) How did you calculate the cost of adverse effects? Please clarify in the Methods section | All expenses were based on the patients' reports, so they were asked about medicines that were purchased, extra consultations and other actions that generated expenses during the treatment of cutaneous leishmaniasis. No costs were calculated from the perspective of the payer (health service). This information has now been clarified in the Methods section, as suggested (Line 185 – 190). |
|  | 8.) Why did you not collect information on comorbidities like hypertension and diabetes, or other infectious diseases? The presence of comorbidities would likely have increased costs, correct? Is there a reason you didn’t you collect that data? | The aim of the article was to collect costs related to the treatment of cutaneous leishmaniasis only, in order to understand the economic impact of the disease during treatment from the patient's perspective. This data on comorbidities was collected (see Table 1) but was not correlated for the dependent variable. |
|  | 9.) Why does Table 4 only report for 61 patients? Is that because 61 out of the 68 patients experienced “catastrophic spending”? Please explain in the document and please relate this to line 278 where you note that “the median expenditure on CL treatment alone reached catastrophic levels for over 40% of families”. Moreover, where in the analysis that you have presented can we see that 40%? | Table 4 shows the average amount spent by patients, separating those with and without catastrophic data. The sample value (n=61) refers to the number of patients who reported this complete data to be analysed in this analysis of associated factors.  The information on catastrophic spending data was presented in the text detailing the number of families and data not reported (Line 266-267). |
|  | 10.) Please add the structured questionnaire that the authors used in the appendix and any details of how costs were calculated for each cost component? It would be useful for other researchers to see what exactly were the cost components that were measured, and how they were measured. | A structured questionnaire was included as a supplementary file. |
